# Population dynamics of ammonium oxidizing and heterotrophic bacteria in a nitrifying consortium fed with ammonium and benzotriazole

**DOI:** 10.1007/s11274-025-04578-2

**Published:** 2025-10-23

**Authors:** María Lynet Aceves-Zamora, Omar Oltehua-López, Flor de María Cuervo-López, Anne-Claire Texier

**Affiliations:** Department of Biotechnology-CBS, Metropolitan Autonomous University Iztapalapa, Av. Ferrocarril San Rafael Atlixco 186, Mexico City, CP 09310 Mexico

**Keywords:** Ammonium oxidizing bacteria, Benzotriazole, Heterotrophic bacteria, Nitrifying consortium, Population dynamics, Sequencing batch reactor

## Abstract

Nitrifying sludge used in biological wastewater treatment contain ammonium oxidizing bacteria (AOB) and heterotrophic bacteria that can enable the simultaneous removal of contaminants. The population dynamics of a nitrifying consortium was assessed in a sequencing batch reactor (SBR) fed with ammonium (cycles 1-130) and benzotriazole (BT) (cycles 40–130). Diversity of the identified AOB and heterotrophs could be associated with the capacity of the sludge for carrying out simultaneously ammonium oxidation and BT biotransformation processes. Thirty-nine OTU’s constituted the bacterial core of the consortium with an abundance higher than 58% of the total population along the experimentation where Nitrosomonadaceae family became dominant during cycles with BT addition. *Nitrosomonas mobilis* and thirteen unassigned OTUs of *Nitrosomonas* genus increased their relative abundance during BT exposure, suggesting their participation in both ammonium oxidation and BT cometabolic biodegradation. The acquirement of metabolic ability of the sludge for biodegrading BT in cycle 47 could also be associated with the highest diversity and richness of species detected during this cycle and the abundance increase of twelve identified heterotrophic bacteria. Results showed that the changes in metabolic capacities of the sludge for carrying out two efficient oxidizing processes reflected the changes in microbial community composition along the SBR operation.

**Graphical Abstract:**

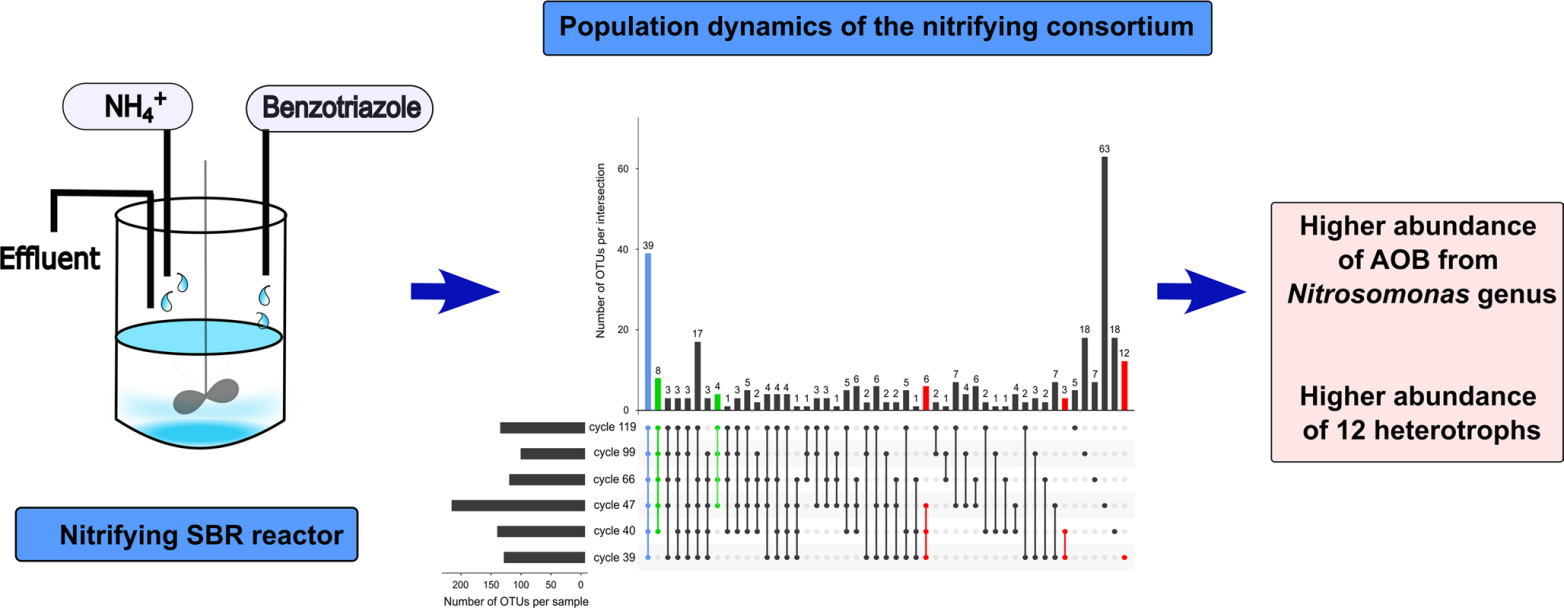

**Supplementary Information:**

The online version contains supplementary material available at 10.1007/s11274-025-04578-2.

## Introduction

Bacterial consortia have been extensively studied and are commonly used in the biological treatment of effluents for their wide and flexible metabolic abilities (Brenner et al. 2008). It has been extensively reported that the whole bacterial community that constitutes the consortium enables the simultaneous biodegradation of various contaminants through the respective metabolic abilities of the different members (Albers et al. 2018). Some authors indicated that bacterial consortia could be designed to treat mixtures of contaminants, such as insensitive munitions compounds, by mixing microorganisms with the required metabolic abilities (Menezes et al. 2022). However, there is also recent and less extensive evidence of the flexibility of the metabolic abilities of microorganisms depending on the feeding conditions under which they are subjected, through processes of enzymatic induction and horizontal gene transfer (Brito 2021; Ramírez-Muñoz et al. 2023). This further expands the possibilities of treatment using consortia (Ferrera and Sánchez 2016). A clear example is nitrifying sludge, where the participation of ammonium oxidizing bacteria (AOB) and heterotrophic microorganisms has been shown in the biodegradation of diverse contaminants from water (Sun et al. 2019; Hernández et al. 2024). The role of lithoautotrophic AOB in the biotransformation of aromatic, phenolic, and halogenated compounds, as well as emerging organic contaminants (EOC) is increasingly demonstrated (Men et al. 2017; Kumwimba and Meng 2019). In many studies, it has been evidenced that the enzyme ammonium monooxygenase (AMO) can play a decisive participation in the oxidation of polluting molecules via cometabolism (Roh et al. 2009; Nzila 2013). Little information is still available on the contribution of AOB in the cometabolic biodegradation of benzotriazole (BT) (Trejo-Castillo et al. 2021). It is also known that heterotrophic bacteria present a wide metabolic capacity for biodegrading organic compounds, even under nitrifying lithoautotrophic conditions (Xu et al. 2018; Sun et al. 2019).

Benzotriazoles are a group of EOC that have been detected in wastewaters and are widely used as anticorrosive and antifreeze additives (Shi et al. 2019; Que et al. 2024). In the case of BT biodegradation by nitrifying aerobic sludge, it has been evidenced that the AMO would be the responsible for its biotransformation under nitrifying lithoautotrophic conditions (Trejo-Castillo et al. 2021). This was shown in 49 d batch cultures but information on cometabolic capacity of the AOB for biodegrading benzotriazoles in dynamic systems is still limited. The continuous or repetitive exposure of the consortium to BT might induce a higher biotransformation by AOB, but also a higher biodegradation by heterotrophs through metabolic adaptation and ecological change processes. A better understanding of wastewater microorganisms involved in the biodegradation of contaminants, as well as their community structure changes linked to varying environmental conditions is needed to improve biological treatment processes (Lydmark et al. 2007; Isobe and Ohte 2014). On the other hand, toxicological effects of benzotriazoles on plants, animals, and microorganisms are more and more documented (Seeland et al. 2012; Shi et al. 2019); therefore, more information on BT effects on bacterial population dynamics of consortia used in wastewater treatment is needed.

Sequencing batch reactor (SBR) systems are non-continuous processes operating in cyclic operational mode where each cycle consists of the fill, react, settle, and draw phases (Singh and Srivastava 2011). The use of SBR systems has been proven to be a successful tool for obtaining multi-functional microbial consortia capable of removing diverse contaminants from wastewater and for propitiating metabolic adaptation of the involved microorganisms (Suárez-García et al. 2019). Previous studies on population dynamics of nitrifying sludge in SBR reactors have shown the enrichment of AOB and heterotrophs throughout the operational cycles, evidencing the decisive role of these microorganisms for biotransforming simultaneously ammonium and organic compounds (Guo et al. 2022; Esquivel-Mackenzie et al. 2024; Martínez-Jardines et al. 2025). More studies using high-throughput sequencing are required to deepen the analysis of community structure/metabolic ability relationships in consortia used for wastewater treatment, particularly in nitrifying sludge.

The objective of this study was to evaluate in a sequencing batch reactor (SBR) the bacterial population dynamics of AOB and heterotrophs of a consortium fed with ammonium and BT.

## Materials and methods

### Nitrifying consortium

The consortium was previously exposed to different mixtures of NH_4_^+^, phenol, cresols (*o*-cresol, *m*-cresol, and *p*-cresol), and sulfide in a SBR reactor for 7.5 months according to the methodology described by Suárez-García et al. (2019). During this previous study, the consortium presented a high ability for ammonium removal with NH_4_^+^ consumption efficiencies values higher than 97.3%. Nitrate was the main product of ammonium oxidation obtained through a catabolic nitrifying process at yields values higher than 0.70 mg NO_3_^−^-N/mg NH_4_^+^-N consumed. This sludge was used for inoculating the SBR reactor at an initial concentration of 350 mg microbial protein/L (equivalent to 0.53 g volatile suspended solids (VSS)/L).

### Sequencing batch reactor

The consortium was fed with ammonium at an initial concentration of 147.3 ± 9.2 mg NH_4_^+^-N/L and BT at 4.74 ± 0.34 mg/L in a 2-L SBR reactor (Applikon, model P100). The composition of the medium was defined for nitrification process as previously reported by Suárez-García et al. (2019). The reactor was operated at 25 ± 3 °C, pH of 8.2 ± 0.2, and continuously shaken (250 rpm) and aerated (dissolved oxygen concentration of 4.0 ± 0.2 mg/L). The operation cycles lasted 24 h and consisted of the following phases: fill (0.08 h), aerobic biological reaction (23 h), settle (0.75 h), and draw (0.17 h). The different SBR phases were controlled electronically by timers. The volumetric exchange ratio of liquid was 85% while the hydraulic retention time was 1.17 d. The SBR was firstly operated under nitrifying conditions without addition of BT for 39 cycles. BT was then added into the reactor from cycle 40 to cycle 130. Samples were withdrawn periodically in the influent and effluent of the reactor, filtered (0.2 μm), and analyzed for ammonium, nitrate, nitrite, and BT. During the biological reaction phase of some cycles, samples of completely mixed liquor were also taken at different times for conducting profiles of BT consumption. The nitrifying capacity of the sludge was evaluated through the following response variables: ammonium removal efficiency (E-NH_4_^+^, %, (g NH_4_^+^-N consumed/g NH_4_^+^-N fed) × 100), nitrate production yield (Y-NO_3_^−^, g NO_3_^−^-N produced/g NH_4_^+^-N consumed), nitrite production yield (Y-NO_2_^−^, g NO_2_^−^-N produced/g NH_4_^+^-N consumed), and biomass production yield (Y-BM, g biomass-N produced/g NH_4_^+^-N consumed). It was considered that 16% of microbial protein is nitrogen for estimating the biomass yield (Bailey and Ollis 1986).

### High-throughput sequencing

The evaluation of the population dynamics was carried out using a control sample corresponding to the nitrifying sludge prior to the addition of BT in cycle 39 (control cycle) and five samples after the addition of BT at cycles 40, 47, 66, 99, and 119. Cycle 40 was chosen for sampling as the first cycle after BT addition. Two samples were taken at cycles 47 and 66 during the transitory phase when nitrification process was altered by BT feeding. Cycles 99 and 119 were selected for sampling as representative of the period when the nitrifying activity was recovered and stabilized. All samples were collected at the end of each cycle. The sludge samples were centrifuged at 5000 rpm for 10 min, the pellets were frozen at −20 °C and stored until DNA extraction was carried out. DNA extraction and purification were performed using the UltraCleanTM Soil DNA Isolation Kit (MO BIO Laboratories, Carlsbad, CA, USA). The integrity of the DNA was evaluated by electrophoresis in agarose gel at 1% (w/v) at 85 V for 40 min. DNA quantification and purity were determined using a microsample spectrophotometer (NanoDrop 2000, Thermo Scientific, USA). Amplification of the V4 region of the 16 S rRNA gene and sequencing were performed by the Beijing Genome Institute (BGI Genomics, Shenzhen, China) using the primers 515F and 806R. The Ilumina HiSeq 2500 paired-ends 250 bp sequencing platform was used for amplicon sequencing.

### Sequencing data analysis

The raw sequencing data were pre-processed by removing the adapter header and barcodes. Low complexity and ambiguous ‘N’ reads with 10 or more same consecutive base were also removed. The analysis of the sequences was carried out with the QIIME 2 (v2020.2) software (Bolyen et al. 2019) using the DADA plugin to eliminate noise. All reads were truncated to 250 pb for analysis. The ARB-SILVA version 138 database was used for the taxonomic assignment using a classifier Naïve Bayes from QIIME 2. The microbial diversity indices were calculated using metric methods available in QIIME 2, the Shannon index was used to represent the alpha diversity, Chao 1 was used to calculate the species richness, and the relative evenness was represented by Pielou’s evenness index. The sequencing data were deposited in the GenBank Sequence Read Archive (https://www.ncbi.nlm.nih.gov/sra/) under BioProject accession number PRJNA1217045.

To create the phylogenetic trees of the sequences, a search for the most similar sequences was carried out by BLAST (https://blast.ncbi.nlm.nih.gov/Blast.cgi) using the NCBI 16 S ribosomal RNA sequence database (Bacteria and Archaea). The sequences of the best hits obtained were used for alignment analysis in M-COFFEE (https://tcoffee.crg.eu/) and PhyML 3.0 softwares to establish the possible phylogenetic relationships. A bootstrap analysis with 1000 iterations was used.

### Analytical methods

BT, nitrate, and nitrite were analyzed by HPLC (PerkinElmer, Series 200). For BT quantification, a C18 reverse phase column (Phenomenex, Bondclone, 3.9 × 300 mm) and a UV detector at 274 nm were employed (Trejo-Castillo et al. 2021). The mobile phase was methanol/water (7:3, v/v) at 1 mL/min. For determination of nitrate and nitrite, an ion exchange column (Waters, IC-Pak Anion HC, 4.6 × 150 mm) and a UV detector at 214 nm were used. The mobile phase was composed of butanol, acetonitrile, and a borate-gluconate solution as previously described by Trejo-Castillo et al. (2021). The flow rate was 2 mL/min. Ammonium nitrogen was measured by a selective electrode (Phoenix Electrode Company) (Suárez-García et al. 2019). Modified Lowry’s method (Lowry et al. 1951; Martinez et al. 2000) and standard methods (APHA 1998) were employed to measure microbial protein concentration and VSS, respectively. pH and dissolved oxygen concentration were determined by selective electrodes. For each method, standard curves were established in triplicates, obtaining coefficients of determination (R^2^) higher than 0.98 and coefficients of variation in the slope value smaller than 2%.

## Results

### Performance of the consortium for nitrifying and biodegrading benzotriazole

**Fig. 1:**
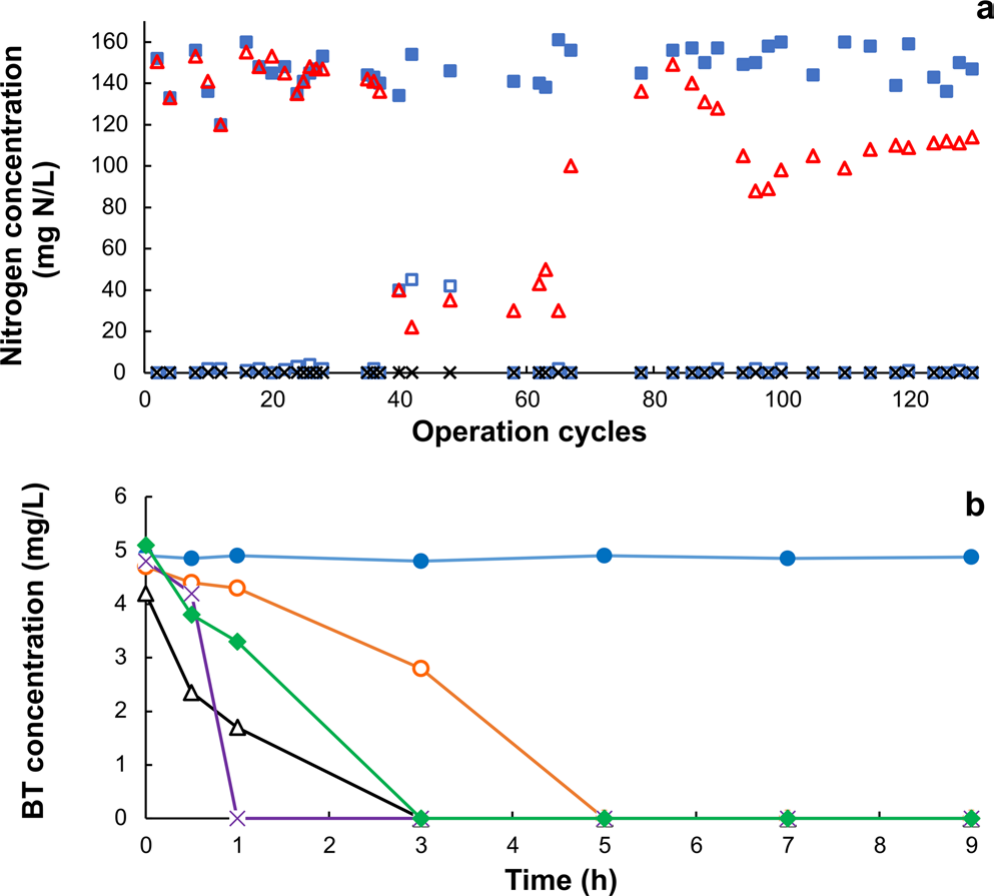
Performance of the consortium for carrying out nitrification and BT biodegradation processes: (**a**) nitrification process in the SBR fed with ammonium (cycles 1–39) and ammonium and benzotriazole (cycles 40–130), () NH_4_^+^-N in the influent, () NH_4_^+^-N in the effluent, (**×**) NO_2_^−^-N in the effluent, () NO_3_^−^-N in the effluent; (**b**) profiles of BT consumption along the operation cycles of the SBR, () cycle 40, () cycle 47, (**Δ**) cycle 66, () cycle 99, () cycle 119

Prior to the addition of BT into the reactor (cycles 1 to 39), most of the consumed ammonium was oxidized to nitrate (Fig. 1a). Nitrite accumulation in the effluent was negligible. Under these conditions, E-NH_4_^+^ was of 99.6 ± 0.3% and Y-NO_3_^−^ of 0.98 ± 0.01 g NO_3_^−^-N produced/g NH_4_^+^-N consumed. The Y-BM was 0.02 ± 0.01 g biomass-N produced/g NH_4_^+^-N consumed. This indicated that the nitrification process was mainly dissimilative and carried out through a catabolic process. These results showed that the consortium performed a highly efficient and stable nitrifying respiratory process for obtaining energy. After BT addition into the SBR, a transitory phase (cycles 40–79) was observed where nitrification activity was altered with ammonium accumulation and a lower production of nitrate. There was no nitrite accumulation in the reactor. From cycle 80 to cycle 130, nitrification performance was recovered, obtaining E-NH_4_^+^ of 99.6 ± 0.3% and Y-NO_3_^−^ of 0.76 ± 0.08 g NO_3_^−^-N produced/g NH_4_^+^-N consumed. These results showed that the consortium was able to adapt to BT exposure as after an alteration in its nitrifying activity, it recovered its metabolic ability to perform a successful nitrification.

During the first cycle of BT addition (cycle 40), the consortium was not metabolically able to biodegrade the EOC which was totally recalcitrant during the entire reaction phase (Fig. 1b). However, seven operation cycles later (cycle 47), it is noticeable how BT was totally consumed in 5 h. During the subsequent cycles from cycle 66 to cycle 119, the performance of the sludge to biodegrade BT was better, obtaining a total consumption in less than 3 h. These results showed that the sludge acquired the capability to biodegrade BT and improved it throughout the SBR operation. This could be related to population dynamics and/or enzymatic induction.

## General composition of nitrifying sludge

**Fig. 2:**
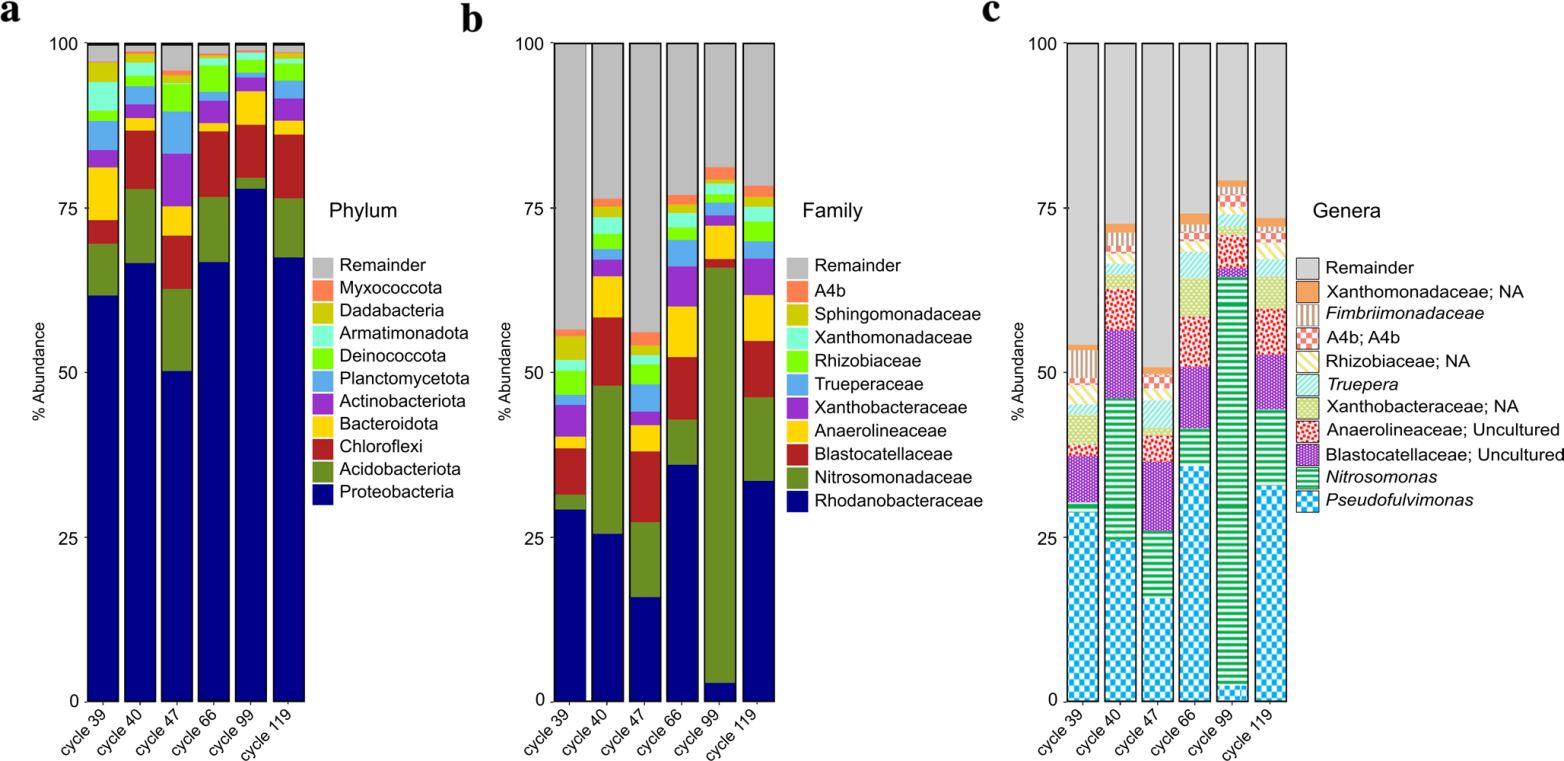
Relative abundance of nitrifying sludge during the operation of the SBR reactor at the (**a**) phylum, (**b**) family, and (**c**) genus levels

The identification of the microorganisms present in the nitrifying sludge was carried out at different taxonomic levels. At the phylum level, Proteobacteria were identified as the dominant phylum in all cycles (cycle 39 = 62%, cycle 40 = 67%, cycle 47 = 50%, cycle 66 = 67%, cycle 99 = 78%, cycle 119 = 68%) (Fig. 2a). Acidobacteriota, Chloroflexi, Bacteroidota, and Actinobacteriota also remained among the most representative phyla despite having changes in their abundance along operation cycles. At the family level, Rhodanobacteraceae was the dominant in five of the six analyzed cycles (Fig. 2b). In cycle 99, Nitrosomonadaceae was the dominant family and always remained within the families with the highest abundance in the other cycles. Its relative abundance increased from 2.2% in cycle 39 without BT to values between 6.8 and 62.9% during BT addition in cycles 40–119, showing a marked increment after BT was introduced. Regarding genera, *Pseudofulvimonas*, *Nitrosomonas*, two uncultured genera of the Blastocatellaceae and Anaerolineaceae families, and an unidentified genus of the Xanthobacteraceae family were the most representative genera throughout the experimentation (Fig. 2c).

**Fig. 3:**
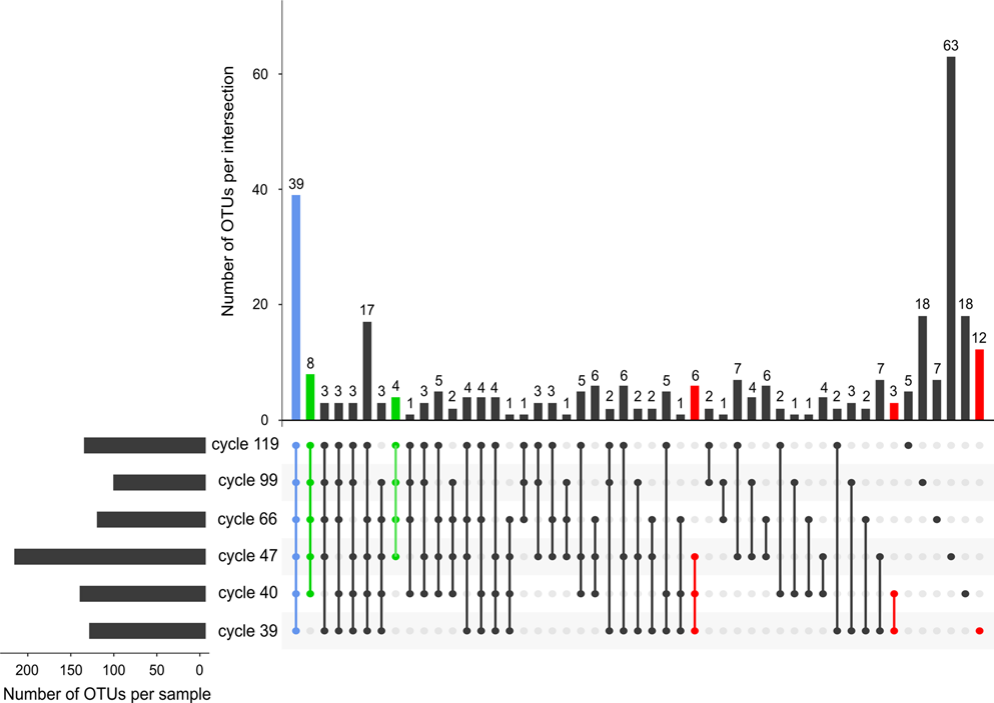
Bacterial population dynamics of the nitrifying sludge during the operation of the SBR reactor. Upset diagram of the frequency of OTUs through the operation cycles. The black circles of the matrix represent the OTUs present in a single SBR operation cycle, the connected black circles indicate the presence of the same OTUs in different operation cycles. The top of bar plot shows the number of detected OTUs per intersection and the lateral bar diagram shows the total number of OTUs identified in each operation cycle. The blue bar indicates the bacteria core of the nitrifying sludge. The green bars indicate the bacteria not detected in cycle 39 but detected from the first cycles of exposure to benzotriazole (cycles 40 or 47) up to cycle 119. The red bars indicate the bacteria present in cycle 39 and up to cycles 40 or 47

At the species level, 315 OTU’s (Operational Taxonomic Unit) were identified as different species throughout all the cycles of operation. In the control cycle (cycle 39), 134 species were identified and in the last analyzed cycle (cycle 119), 140 species were detected (Fig. 3). The cycle with the highest number of species detected was cycle 47 with 221 and the least number of species was detected in cycle 99 with only 106. These results are reflected in the Shannon diversity and Chao 1 richness analyses, indicating that in cycle 47 there was a greater diversity and richness of species (Table 1). In the same way, the less diversity of species and the lowest value of Chao 1 richness were registered in cycle 99. The Pielou’s evenness index ranged between 0.822 and 0.873 in all cycles except in cycle 99, showing stability in the evenness of the consortium. This coincides with the slight changes observed in the abundance of microbial populations in the different cycles of operation (Fig. 2c). However, in cycle 99, the Pielou’s evenness index dropped to 0.690, indicating a higher predominance of species over others. This is related to the higher abundance of *Nitrosomonas* in this cycle.

**Table 1:** Indices of microbial diversity, richness, and uniformity in different SBR operation cycles

Cycle	Shannon diversity	Chao 1 richness	Pielou’s evenness
39 40 47 66 99 119	6.648 6.290 7.323 6.311 5.094 6.391	213 200 335 199 167 219	0.860 0.823 0.873 0.826 0.690 0.822

### Bacterial core and population dynamics

To identify the core of bacteria that made up the nitrifying sludge, a search was carried out for the species that were detected in all operation cycles, showing that 39 different OTU’s constituted the core (Fig. 3). Despite BT exposure, the sludge core remained above 58% of abundance in all cycles (Online Resource 1).

**Fig. 4:**
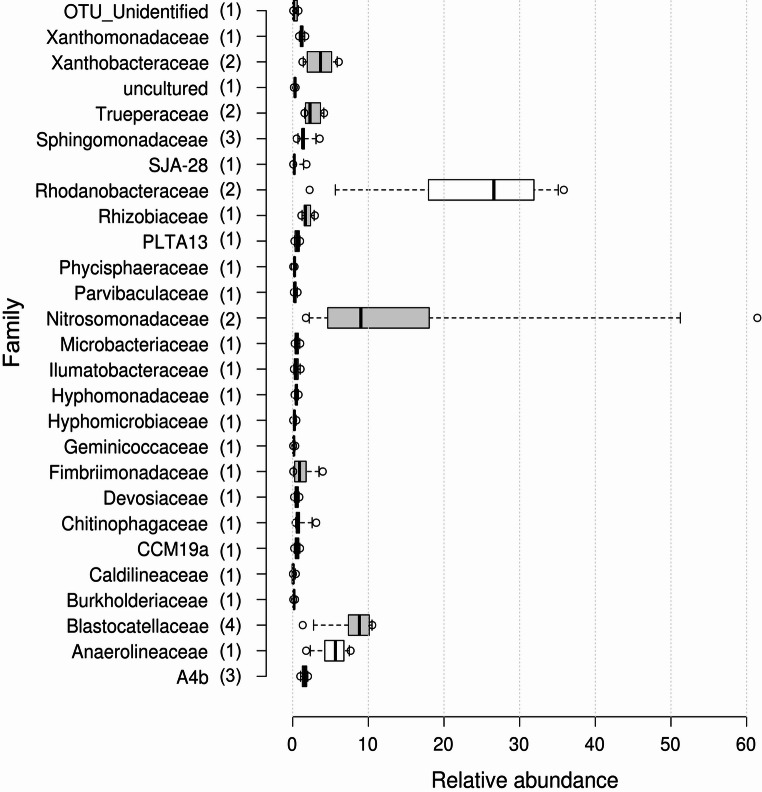
Relative abundance of the families of bacteria that make up the core of the nitrifying sludge during the cycles of exposure to benzotriazole. Center lines show the medians; box limits indicate the 25th and 75th percentiles as determined by R software (BoxPlotR; http://shiny.chemgrid.org/boxplotr/); whiskers extend to 5th and 95th percentiles, outliers are represented by dots. *n* = 5, corresponds to the sample points (cycle 40 to 119)

It was possible to identify at species level 38 OTU’s of the core. They belong to 26 different families, wherein six families were dominant: Rhodanobacteraceae 26.6%, Nitrosomonadaceae 9.0%, Blastocatellaceae 8.8%, Anaerolineaceae 5.6%, Xanthobacteraceae 3.7%, and Trueperaceae 2.3% (Fig. 4). Before BT exposition, the dominant families of the core with an abundance percentage greater than 3% were: Rhodanobacteraceae 28.9%, Blastocatellaceae 7.0%, Xanthobacteraceae 4.8%, Fimbriimonadaceae 3.9%, Sphingomonadaceae 3.6%, and Chitinophagaceae 3.1%. However, in the first cycle of BT exposition, the families Fimbriimonadaceae, Sphingomonadaceae, and Chitinophagaceae decreased in abundance to 2.0%, 1.6%, and 0.4%, respectively. In subsequent cycles, none of these families exceeded 1.6% of abundance. On the other hand, the Nitrosomonadaceae and Anaerolineaceae families increased their abundance after exposure to BT from 1.7% (cycle 39) to 20.0% (cycle 40) and 1.7–6.2%, respectively. The Nitrosomonadaceae family was the second most dominant of the nitrifying sludge core during exposure to BT (Fig. 4). The Trueperaceae family managed to increase its abundance until cycle 47 from 1.5 to 4.0%, maintaining its position as the sixth most dominant family in the cycles with exposure to BT and preserving an average of 2.3% after cycle 47 (Fig. 4).

**Fig. 5:**
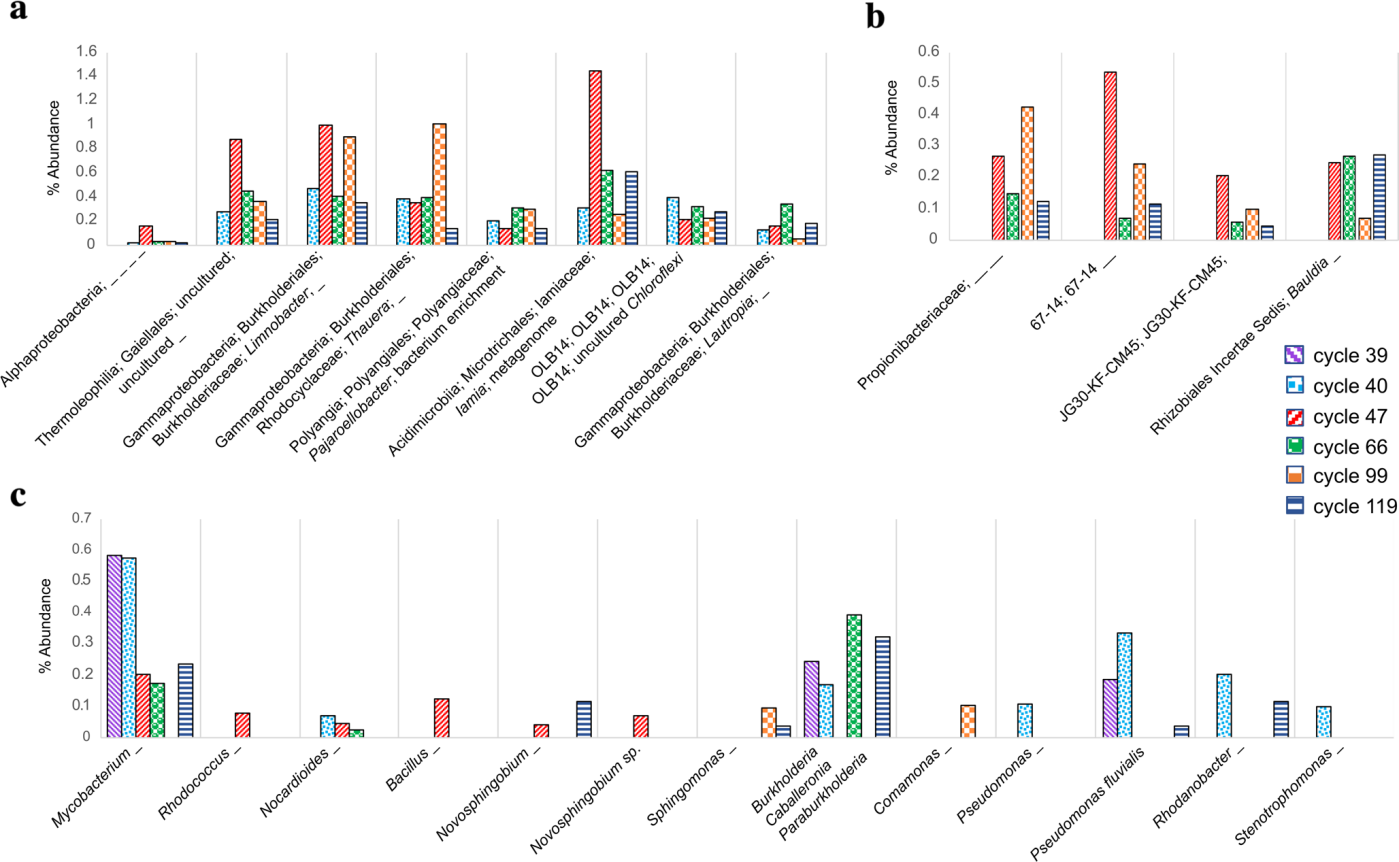
Relative abundance of heterotrophic bacteria prior to the addition of benzotriazole (cycle 39) and during different cycles of SBR operation with BT feeding (cycle 40 to cycle 119). (**a**) Bacteria detected since cycle 40 after BT feeding and that remained up to cycle 119. (**b**) Bacteria detected since cycle 47 and that remained up to cycle 119. (**c**) Bacteria previously reported in the literature as detected in media with hydrocarbon compounds

According to Fig. 3, some bacteria were favored (green bar) or sensitive (red bar) to BT exposure. In this sense, eight bacteria were detected from the first exposure cycle (cycle 40) and remained present in subsequent cycles. These eight species were identified as heterotrophs belonging to the genera *Limnobacter*,* Thauera*, *Pajaroellobacter*, *Iamia*, OLB14, *Lautropia*, an uncultured genus from order Gaiellales, and an OTU from class Alphaproteobacteria (Fig. 5a). Other four bacteria were detected since cycle 47 and were able to remain present in the consortium up to cycle 119 (Fig. 3). These four OTU’s were identified as heterotrophic bacteria belonging to the genera 67 − 14 (order Solirubrobacterales), JG30-KF-CM45, *Bauldia*, and a member of the Propionibacteriaceae family (Fig. 5b). These twelve species would represent the heterotrophic bacteria favored by exposure to BT.

Meanwhile, other twelve identified bacteria belonging to the genera SH-PL14, *Legionella*, *Gemmatimonas*, *Steroidobacter*, Candidatus_Nucleicultrix, *Terrimonas*, WPS-2, an uncultured genus from Pirellulaceae family, an uncultured genus from order Microtrichales, an uncultured genus from phylum Armatimonadota and two bacteria from the Microscillaceae and Chitinophagaceae families resulted to be BT sensitive species as they were only detected in cycle 39 prior to BT exposure (Fig. 3). Likewise, three bacteria of the genera *Flavobacterium*, *Fluviicola*, and one species of the Reyranellaceae family, as well as six identified bacteria belonging to the genera *Planctomicrobium*, *Legionella*, *Mesorhizobium*, SM1A02, AKYG1722, and a member of the Holosporaceae family, were sensitive to exposure to BT, as they were only detected up to cycles 40 or 47, respectively.

### Population dynamics of ammonium oxidizing autotrophic bacteria

**Fig. 6:**
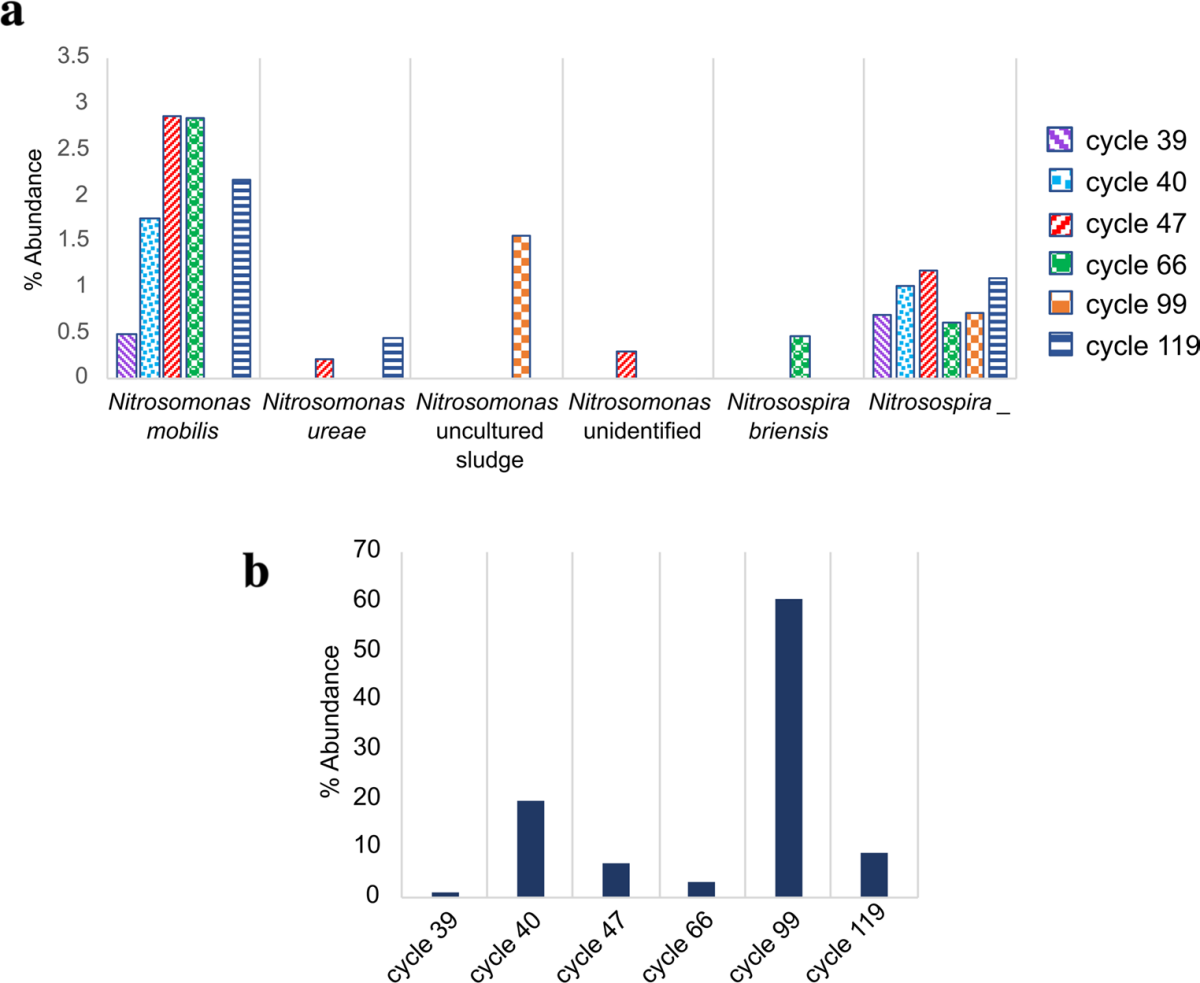
Relative abundance of AOB prior to the addition of benzotriazole (cycle 39) and during different cycles of SBR operation with BT feeding (cycle 40 to cycle 119). (**a**) Ammonium oxidizing autotrophic bacteria abundance. (**b**) Most abundant unassigned AOB species of the genus *Nitrosomonas* (N1-N13)

In the group of AOB present in the nitrifying sludge, the *Nitrosomonas* and *Nitrosospira* genera were identified (Fig. 6a). Both genera were present in all operation cycles but not all species remained present in all cycles. As previously observed (Fig. 2c), *Nitrosomonas* was the second most abundant genus detected in BT exposure cycles. In the *Nitrosomonas* genus, *Nitrosomonas mobilis* was identified in five of the six cycles with an abundance greater than 1.7% during the cycles with exposure to BT and less than 0.5% in the control cycle. *Nitrosomonas ureae* was detected in cycles 47 and 119 with an abundance lower than 1% while two other unidentified species of the genus *Nitrosomonas* were detected in one cycle (*Nitrosomonas* uncultured sludge and *Nitrosomonas* unidentified). To taxonomically assign these species within the genus *Nitrosomonas*, the assembled sequences of both groups N_Un (*Nitrosomonas* unidentified) and N_us (*Nitrosomonas* uncultured sludge) were used to make a BLAST using the 16 S ribosomal RNA sequences database (Bacteria and Archaea) from NCBI (Online Resource 2, Table S1). The OTU identified as N_Un is most closely related to *Nitrosomonas aestuarii* while the OTUs of the N_us group are related to *Nitrosomonas europaea*. Furthermore, during the taxonomic assignment, it was not possible to assign thirteen OTUs (N1-N13) among the different characterized species of the genus *Nitrosomonas* from the SILVA database (Online Resource 2, Table S2). However, these OTUs maintained the highest abundance in all cycles within the genus *Nitrosomonas* (Fig. 6b). Prior to exposure to BT in the control cycle, these OTUs only reached 1% of abundance in the nitrifying sludge but after exposure to BT (cycles 40 to 119), their abundance was higher and reached its greater value in cycle 99 with more than 60% of the total population. As observed in Fig. 6b, the relative abundance of these thirteen OTUs decreased from cycle 40 to cycle 66. This coincides with the transitory phase (cycles 40–79) observed in the reactor where nitrification activity was altered. The higher abundance of these OTUs obtained in cycle 99 might be related to the recovery of metabolic function enabling effective nitrification (cycles 80–130). These results indicate a key contribution of these thirteen unassigned AOB species of the genus *Nitrosomonas* (N1-N13) in the ammonium oxidizing process. Additionally, the sequences of the best hits obtained using BLAST (Online Resource 2, Table S2) were used for the alignment analysis in M-COFFEE (https://tcoffee.crg.eu/) and PhyML 3.0 softwares to establish the possible phylogenetic relationships (Online Resource 3). Although the values ​​supporting the correlation were low, the results suggested that eleven of the thirteen OTUs are more associated with the species *Nitrosomonas europaea*, while N10 is associated with *Nitrosomonas mobilis*. Interestingly, N3 seems to be more related to the genus *Nitrosospira*, however the sequence size used greatly limited the association due to the high sequence similarity in both genera. This suggests that this group of thirteen OTUs belonging to the family Nitrosomonadaceae might be species that have not been previously reported, although the analysis of the V4 region is limited and further analysis is required.

In the genus *Nitrosospira*, *Nitrosospira briensis* was only detected in cycle 66 with an abundance of 0.46% (Fig. 6a). Another two OTUs were detected, however, it was not possible to assign them to any reported species of the *Nitrosospira* genus from the SILVA database. These OTUs remained present in all cycles with an abundance no more than 1.2%.

### Population dynamics of heterotrophic bacteria

Regarding the heterotrophic bacteria present in the sludge, as previously described, twelve identified heterotrophs were favored by BT exposure (Fig. 5a and b). The group of eight bacteria that were detected since cycle 40 maintained as a whole, a relative abundance of 2.2% in total in cycle 40, with a higher total abundance of 4.3% in cycle 47 when the complete consumption of BT was carried out (Fig. 5a). In the same way, it is possible that the four heterotrophic species detected since cycle 47 and during all subsequent cycles would also be involved in the BT biodegradation process (Fig. 5b). The highest overall abundance of these four heterotrophic bacteria was observed in cycle 47 with a value of 1.2% in total, followed by cycle 99 with 0.8% of total abundance.

A search was made for identifying species or genera previously reported in the literature as associated with the BT biotransformation process or capable of growing in media with hydrocarbon compounds (Fig. 5c). Thirteen different species belonging to eleven different genera were identified in the sludge. The most frequent and abundant detected species were related to the *Mycobacterium* genus, followed by species of the genus *Burkholderia-Caballeronia-Paraburkholderia*. It is important to note that these species did not exceed 0.6% of individual abundance in each cycle. On the other hand, in the first exposure cycle to BT (cycle 40), the abundance of species of the genera *Nocardioides*, *Pseudomonas*, *Rhodanobacter*, and *Stenotrophomonas* was increased. While in cycle 47 the increase in abundance of the genera *Rhodococcus*, *Bacillus*, and *Novosphingobium* was favored. However, despite their abundance increased, these genera were not detected in all subsequent cycles. The genera *Novosphingobium*, *Sphingomonas*, *Pseudomonas*, and *Rhodanobacter* were detected in the last operation cycle (cycle 119) with an abundance that did not exceed 0.2%.

## Discussion

The consortium used in the SBR reactor was able to simultaneously biodegrade ammonium and BT. The whole bacterial community of the sludge presented high diversity and richness since cycle 39 without BT addition and up to the end of experimentation during addition of the EOC. The widespread bacterial diversity could allow to sustain metabolic abilities for both ammonium oxidizing and BT biodegrading processes, including diverse AOB and heterotrophic bacteria. The stability in the evenness of the bacterial communities along the operation cycles might also be crucial for performing both oxidizing processes (Xun et al. 2019; Chang et al. 2023). Despite the bacterial communities presented similar indices of diversity, richness, and evenness at cycles 39 and 119, results showed changes in the microbial populations composition. These changes in the bacterial communities of the sludge occurred during all the operation period of the SBR with modifications in the detected bacteria as their abundance. These modifications in the bacterial populations were partly related to their abilities to use ammonium or BT as energy sources for growth, but also to their sensitivity to the BT as some bacteria were not able to remain detectable after BT addition to the reactor, probably linked to toxic effects of BT (Pillard et al. 2001; Shi et al. 2019). Thirty-nine OTU’s constituted the core of the consortium with an abundance higher than 58% of the total population along the experimentation. The dominant families suffered changes in abundance with the addition of BT. Some families such as Nitrosomonadaceae, Anaerolineaceae, and Trueperaceae were favored after BT exposure. Other families such as Fimbriimonadaceae, Sphingomonadaceae, and Chitinophagaceae decreased in abundance.

Ammonium oxidation could be associated with the presence of AOB from Nitrosomonadaceae family. The genera of *Nitrosomonas* and *Nitrosospira* remained present during all the operation cycles, but their species changed along the cycles. *Nitrosomonas* was one of the most abundant genera throughout the experimentation. Some AOB from *Nitrosomonas* genus increased their abundance after BT exposure such as *Nitrosomonas mobilis* and thirteen unassigned OTUs of *Nitrosomonas*. These thirteen AOB maintained their abundance between 2.9% and 60.6% along the SBR operation with BT addition. These results indicated that these bacteria were able to oxidize ammonium in the presence of BT, but also suggested that they could participate in BT biodegradation (Canto-Encalada et al. 2022; Chawley and Jagadevan 2023).

The acquirement of metabolic ability of the sludge for biodegrading BT in cycle 47 might be associated with the highest diversity and richness of species found during this cycle. Metabolic adaptation for better BT biodegradation along the cycles could be related to the presence of heterotrophic bacteria able to consume BT such as the twelve identified heterotrophic OTUs favored by exposure to BT, appearing since the first exposure cycles 40 or 47 and remaining up to the end of experimentation. Moreover, the highest overall abundance (5.5%) of these twelve heterotrophic bacteria was observed during cycle 47 when the total BT removal started to be observed in the reactor. It cannot be discarded that some AOB present in the consortium might also contribute to oxidize the EOC as it has been previously shown that the enzyme AMO was involved in the cometabolic biotransformation of BT (Trejo-Castillo et al. 2021). Actually, during exposure to BT, the Nitrosomonadaceae family was favored in abundance and became the second most dominant family of the sludge core. Cometabolic capacities of AOB can lead to the biotransformation of contaminants without using them as growth-substrates while ammonium is the main energy source (growth-substrate) (Nzila 2013). On the other hand, some heterotrophs can have the metabolic abilities to use BT as energy and carbon source, allowing synergistic removal of the contaminant from water. Thus, the capacity of the consortium for biotransforming BT could be related to its diversity and richness in heterotrophs and AOB.

From the thirteen heterotrophs previously reported in the literature as found in media with hydrocarbon compounds, including BT, *Mycobacterium* and *Burkholderia-Caballeronia-Paraburkholderia* were the most frequent and abundant genera along the cycles. Pathogenic species of *Mycobacterium* such as *M. tuberculosis* and *M. leprae* were not found in the consortium. Interestingly, some BT derivatives have been used to counteract the growth of these species (Briguglio et al. 2015). There are other species of this genus such as *M. cosmeticum* capable of degrading aromatic compounds such as benzene (Zhang et al. 2013). While the genus *Burkholderia-Caballeronia-Paraburkholderia* is involved in the fixation of nitrogen associated with the roots of plants, promoting growth, there are also some species of this genus that have been reported as tolerant to the presence of benzene (de León-Martínez et al. 2017). From the previously reported genera that increased in abundance at cycle 40 when BT was added, some strains of *Nocardioides*, *Pseudomonas*, *Rhodanobacter*, and *Stenotrophomonas* have been shown to be able of growing in hostile environments with aromatic compounds such as *Stenotrophomonas maltophilia* T3-c and *Rhodanobacter lindaniclasticus* RP5557 (Lee et al. 2002; Green et al. 2012; Nogales et al. 2017). *Nocardioides sp.* strain S514 has been reported to be able to degrade BT (Jog et al. 2021). While from the previously reported genera that increased in abundance at cycle 47, *Rhodococcus pyridinovorans* has been associated with the biodegradation of azoles such as benzothiazole, *Bacillus* species with benzene biodegradation, and *Novosphingobium* with its adaptation to environments contaminated by aromatic compounds (Haroune et al. 2002; Liu et al. 2010; Notomista et al. 2011).

## Conclusions

In summary, the whole bacterial community of the nitrifying consortium integrated AOB and heterotrophs that enabled the simultaneous processes of ammonium oxidation and BT biodegradation. The population dynamics was probably related to the capacities of the bacteria to use ammonium or BT as energy sources for growth as their sensitivity to the BT toxicity. The core of 39 OTU’s present in the consortium from the first to the last operation cycle represented more than 58% of the total population along the experimentation, wherein Rhodanobacteraceae was the predominant family. Nitrosomonadaceae increased in abundance after BT addition, becoming the second most dominant family of the bacterial core. The high capacity of the consortium for biotransforming BT could be related to its widespread diversity and richness in heterotrophs and AOB. Twelve identified heterotrophic OTUs favored by exposure to BT could be associated with the capacity of the sludge to biodegrade BT. *Nitrosomonas mobilis* and thirteen unassigned OTUs of *Nitrosomonas* increased their abundance after BT addition, indicating that these bacteria could be involved in ammonium and BT oxidizing processes, besides that they were able to remain in the consortium despite BT exposure.

## Supplementary Information

Below is the link to the electronic supplementary material.

Supplementary Material 1 (PNG211 KB)

Supplementary Material 2 (XLSX 19.3 KB)

Supplementary Material 3 (PNG 327 KB)

## Data Availability

Data supporting the findings of this study are included in this article and openly available in supplementary information files. Should any raw data files be needed in another format they are available from the corresponding author upon reasonable request. The sequencing data were deposited in the GenBank Sequence Read Archive (https://www.ncbi.nlm.nih.gov/sra/) under BioProject accession number PRJNA1217045.
