# Supplementary figures and images for: Population dynamics of ammonium oxidizing and heterotrophic bacteria in a nitrifying consortium fed with ammonium and benzotriazole
Source: World J Microbiol Biotechnol. 2025 Oct 23;41(10):398. doi: 10.1007/s11274-025-04578-2 (PMC12549744; doi:10.1007/s11274-025-04578-2)

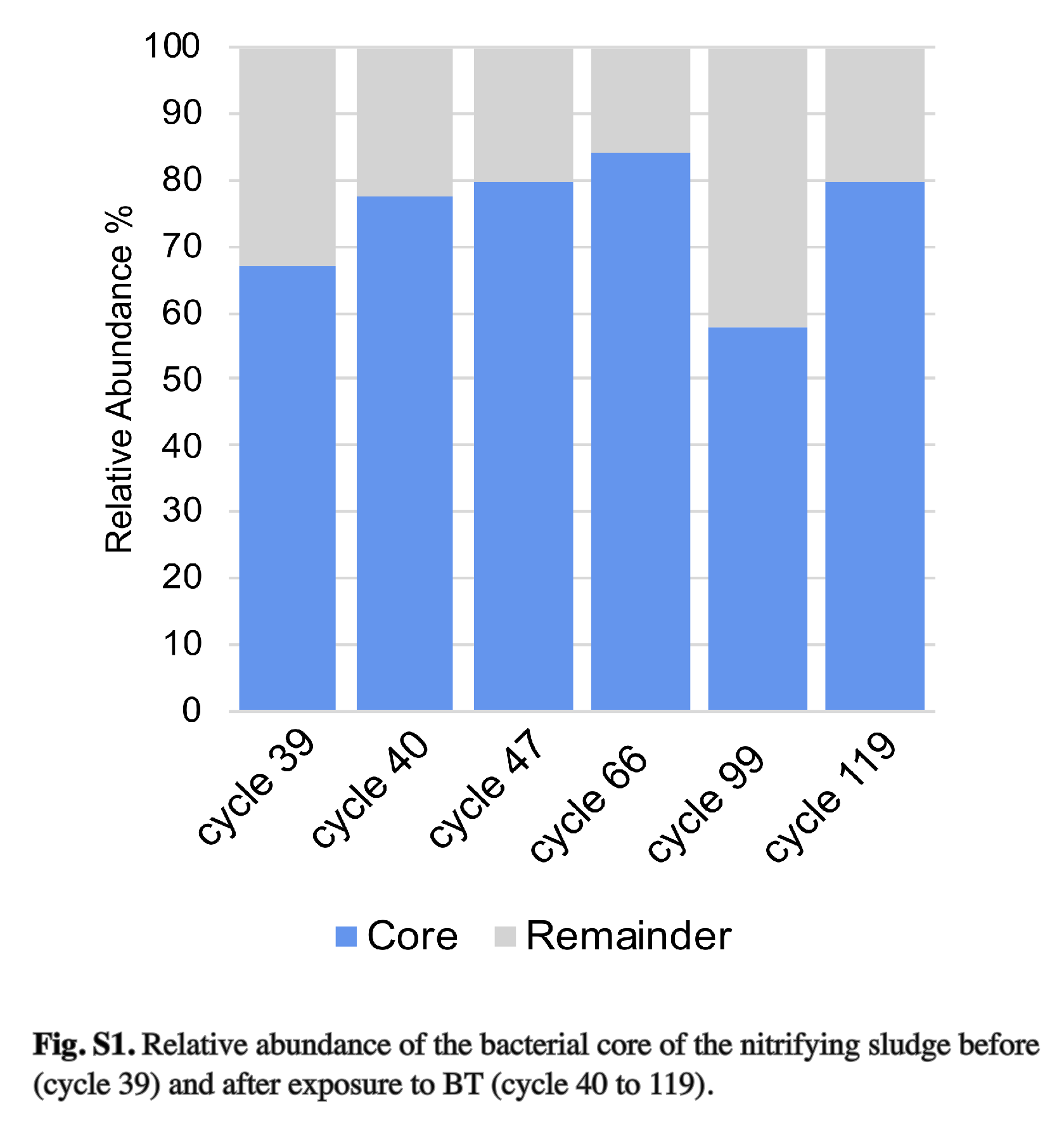

Supplement: Supplementary file 1 — Supplementary Material 1 (PNG211 KB) [file 11274_2025_4578_MOESM1_ESM.png]

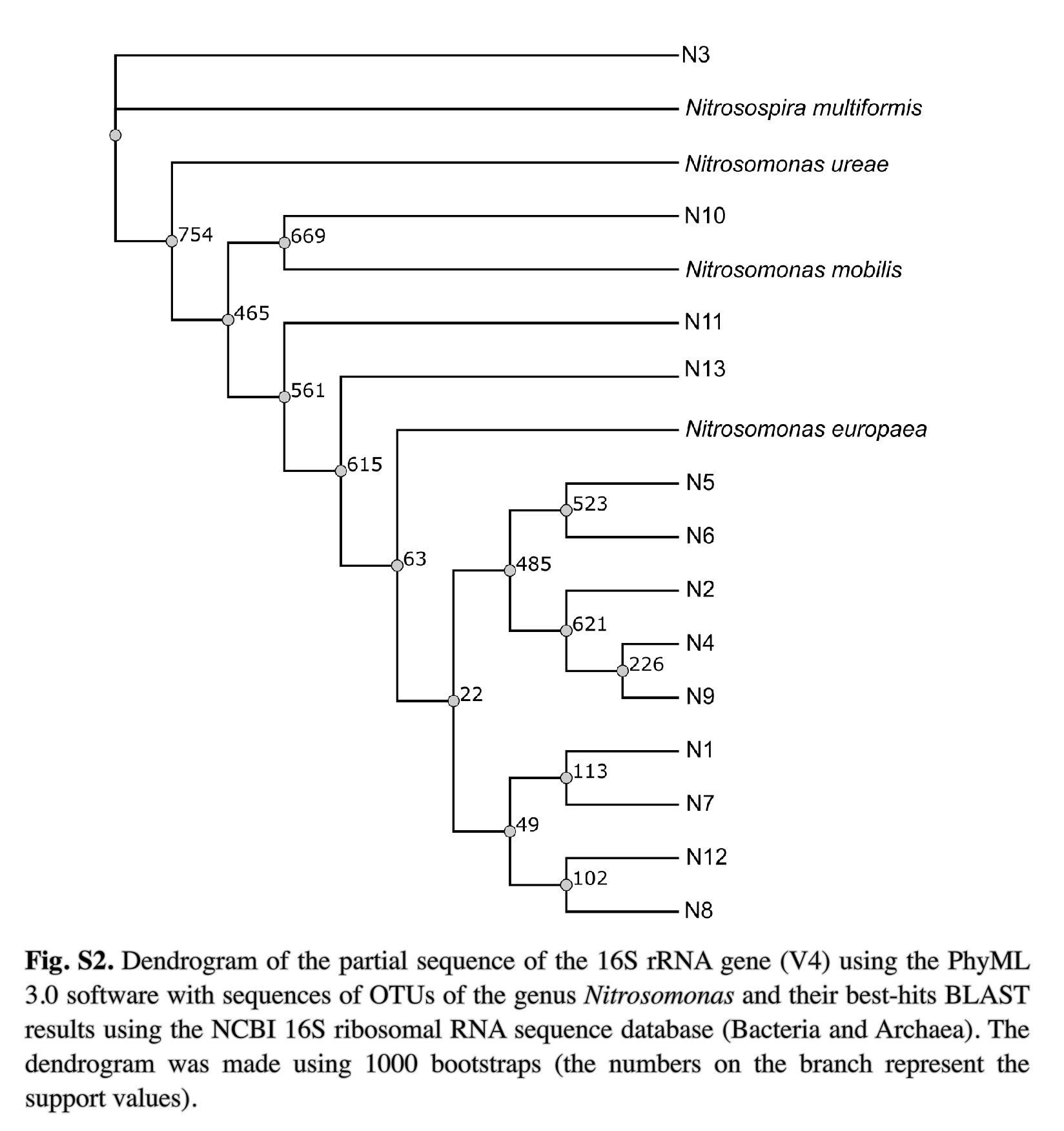

Supplement: Supplementary file 3 — Supplementary Material 3 (PNG 327 KB) [file 11274_2025_4578_MOESM3_ESM.png]
